# Treating a Case of First-Episode Psychosis to Uncover Undiagnosed Obsessive Compulsive Disorder: A Case Report

**DOI:** 10.7759/cureus.41184

**Published:** 2023-06-30

**Authors:** Larayeb Wazir, Dario A Astacio, Suzanne M Lind

**Affiliations:** 1 Psychiatry and Behavioral Sciences, Army Medical College, National University of Medical Sciences, Rawalpindi, PAK; 2 Psychiatry, St. Mary's General Hospital, Passiac, USA; 3 Child and Adolescent Psychiatry, St. Mary's General Hospital, Passiac, USA

**Keywords:** ocd/ anxiety disorders, suicide attempt, insight, first episode psychosis, obsessive compulsive disorders

## Abstract

Obsessive-compulsive disorder (OCD) is a chronic psychiatric condition characterized by the presence of obsessions, compulsions, or both. Historically OCD was associated with good insight. However, there are more categories to the degrees of insight in OCD patients, namely good, fair, poor, absent, or delusional beliefs. It is also important to note that insight can fluctuate circumstantially. We describe a rare case of first-episode psychosis of undetermined cause presenting with suicidal ideation. After continued treatment, it was discovered to be previously undiagnosed OCD with poor insight.

## Introduction

Obsessive-compulsive disorder (OCD) is characterized by obsessions such as recurrent or persistent thoughts, images, urges, and compulsions; repetitive behaviors or mental acts that are aimed at preventing or reducing stress caused by obsessions. Individuals may attempt to ignore, suppress, or neutralize obsessions with compulsions. The lifetime prevalence of OCD is reportedly in the range of 1.5% to 3.5% [1]. Historically, OCD was associated with good insight [2]. However, the Diagnostic and Statistical Manual of Mental Disorders, 4th Edition (DSM IV) field trial first showed that a quarter of the participants were uncertain about whether their symptoms were unreasonable or excessive [3], and this brought attention to the possibility of ‘insight on a spectrum’. The degree of patient insight into the disruptive nature of the symptoms has been more recently studied [4]. The Diagnostic and Statistical Manual of Mental Disorders, 5th Edition, Text Revision (DSM-5 TR) addresses three categories of insight: good, fair, poor or absent insight, or delusional beliefs. The degree to which a patient can recognize the unreasonable and distressing nature of the beliefs helps make the distinction between these categories. 'Poor insight' can function as a specifier for a patient subgroup on the more severe end of the OCD spectrum [5]. In addition to this, de novo OCD was associated with the development of mood disorders with psychotic features and psychotic disorders not otherwise specified (PDNOS) [6]. A complex clinical presentation, diminished response to standard pharmacological interventions, and a poorer prognosis were more prevalent in patients with poor insight, and current therapeutic guidelines recommend the use of different drugs for management, including antipsychotics [7]. It is also important to note that insight can fluctuate with treatment and along the course of the psychiatric condition [8].

## Case presentation

A 31-year-old female was brought into the ER by her husband after he found her in the kitchen holding a knife to her wrist earlier in the day. This was her third visit to the ER in the span of one month. Both of her previous visits had been for similar complaints: increasing anxiety, restlessness, and sleeplessness. In these encounters, she reported feeling worsening anxiety after starting a new and stressful paralegal job. The patient considered the stress to be unbearable, and she subsequently quit. Her first visit to the ER had been on a Saturday, after which she was medically cleared and placed on a psychiatric consult. The assistant nurse practitioner (APN) recommended discharge with a prescription of 2 mg lorazepam and an outpatient behavioral health clinic follow-up. Unfortunately, the outpatient clinic was closed on the weekends, and the patient was asked to return to the ER if her symptoms worsened before Monday. 

Less than 48 hours later, the patient had her second ER visit. Once again, she was medically cleared and discharged with the recommendation to attend the scheduled intake interview with the outpatient clinic. The following day at the outpatient clinic, the APN diagnosed her with anxiety. The patient was prescribed buspirone 5 mg (twice daily) and sertraline 50 mg (once daily), as well as a weekly follow-up with the APN and a therapist separately. Despite being compliant with this regime for a month, she was found by her husband holding a knife to her wrist. He decided to stay with her to make sure she was safe. Luckily, she had another outpatient session scheduled for that day. This incident was reported to the APN, who deemed it necessary for her to go to the ER once more for what appeared to be an attempted suicide event. 

In the ER, the case was further explored with a psychiatric evaluation. The interviewer noted negative symptoms such as no eye contact, no facial expressions, and no intonation of speech. The mental status evaluation revealed that although the patient was alert and oriented, she was very restless and exhibited disorganized thinking, bizarre behaviors, blunted affect, and a dysphoric mood. The patient reported being unable to sleep for more than two hours for the past two months. She had said that sections of her brain were ‘fried’ since she started the psychotropic medications. She had difficulty remembering things and was very preoccupied about not getting better with the medications. In between her responses and while answering questions, the patient was constantly pulling at the ends of her hair, picking her nails and the skin on her legs. She was asked about these behaviors, to which she replied, "I just do it when I feel anxious." When asked if these acts provided relief from stress, the patient was unable to respond. She denied having any auditory or visual hallucinations. 

Collateral information was obtained from the patient's husband. He reported that her symptoms began increasing gradually day by day over the past week. She had been pacing and was only sleeping for about two hours at night. She was also picking at her skin, nails, and hair more often. He had also noted more erratic behaviors that she had never done before. He shared that "she chewed through two shirts and through the bed sheet" and "has been biting her cheek severely to the point where there is a massive wound on the inside." It was agreed that she would benefit from inpatient admission for safety, and the patient was voluntarily admitted. 

On the first day of admission, she continued to have uncomfortable thoughts about wanting to cut herself with a knife. She remained noticeably anxious and showed fidgety behavior; picking her nails and pulling at her hair constantly during the psychiatric evaluation. When asked about these behaviors, she said that they made her "feel more in control." The chart review showed that the patient had been on sertraline 50 mg for one month. A decision was made to increase the dose to 100 mg (once daily) and add aripiprazole 5 mg (once daily). The next day, she reported that she had not been sleeping well since her admission. It was the psychiatrist’s observation that the patient was experiencing thought-blocking in response to open-ended questions during the interview. She responded to the questions trying to ascertain thought content by saying, "I can't think clearly." The patient exhibited fidgety behavior, interrupted speech, and had difficulty expressing her thoughts. She did not make eye contact and wrung her hands constantly. A bilateral tremor in both her upper extremities was also noted. Propranolol extended release 60 mg (every morning), trazodone 50 mg (at bedtime), and Klonopin 0.5 mg (twice daily) were subsequently added. The next day, she noted an improvement in her sleep and her mood. She reported being able to enjoy watching television and participating in therapy sessions. The patient endorsed that it was the first time she had felt like she "was thinking clearly again". It was the nursing staff's and recreational therapist’s observation that she was becoming slightly more interactive. In the subsequent interview, she reported daytime sleepiness, so aripiprazole was adjusted to be given at bedtime and buspirone was discontinued. 

An in-depth psychiatric interview was conducted to establish a definitive diagnosis. It was during this interview that the patient recounted a long-standing history of her fixation on balance and evenness. She said that she always had to count the number of times she chewed food on each side of her mouth and balance it. She shared that this balancing made her feel more in control. She had never been formally diagnosed with OCD before. Her anxiety began to worsen once she started working at a stressful job, and quitting her job curtailed the stress temporarily. It was suspected that the prescribed medications, initial encounters, and outpatient therapy could not bring about any substantial improvement in her case because the full depth of the condition was underexplored. Additionally, buspirone probably exacerbated her anxiety. She had developed intrusive, derogatory thoughts and suicidal ideation. The discomfort caused by her thoughts was considered one of the triggers for the development of suicidal ideation. After this evaluation, the diagnosis of OCD with PDNOS was finally established. The Yale-Brown Obsessive-Compulsive Scale (YBOCS) score was 38 (32-40: extreme OCD) and the Brown Assessment of Beliefs Scale (BABS) showed a score of 21 (≥18 plus a score of 4 on item 1: absent insight/delusional beliefs). 

Throughout the remaining three days of her admission, the patient continued to show improvement. She was discharged home with a referral to a partial hospitalization program (PHP)/intensive outpatient program (IOP) to help continue her recovery, manage her medication, and develop coping skills. She attended PHP for four weeks and the IOP for six weeks. On the day prior to her discharge from the program, a follow-up psychiatric interview was conducted over the phone. She recounted that in the days leading up to her admission, her worsening anxiety coupled with body-picking compulsions had made her feel trapped. She felt unable to communicate with people and had difficulty remembering the names of things that she previously knew. At one time, she even found herself unable to comprehend the words on a screen. Before this, she had been a high-functioning member of society, but over time, her functionality began to decline. She was unable to manage her time, cook, or drive.

It was only after therapy and self-reflection that the patient was able to identify the delusions she had been believing. This was further explored, and the patient said that at the time she was convinced that she and her husband "were going to go broke", "would lose their land," and "die penniless." She confirmed that the beliefs were unfounded as her husband was still employed and they were not, in fact, spending over their budget. She also reported that, at times, she would think that her husband was delusional for not believing her. She shared that she was not sure of exactly what "disease" she had but was convinced that she "would die because of her health", "would never sleep again," and would "die before her parents." These thoughts were unwanted and uncomfortable, but she had the full conviction that they were true. Although she had never believed that the individuals at her workplace were trying to harm her or were out to get her, she was convinced that her job was the cause of her symptoms. She maintained that putting a knife on her wrist was not a compulsion due to her OCD but rather a contemplation of a suicide attempt. She knew, however, that she would not harm herself. 

Her YBOCS score after completing the therapy program was 12 (8-15: mild OCD) and her BABS score was 13 (13-17: poor insight). At this follow-up interview, approximately three months after her inpatient discharge, she reported being on a regimen of sertraline 100 mg (once daily) and propranolol extended-release 60 mg (once daily). Eighteen days after her discharge from the inpatient unit, aripiprazole was reduced to 2.5 mg (once daily), and the patient continues to take this dose. She is able to get five to nine hours of sleep without needing medications. The patient reports that she now feels happier and more hopeful. The program has helped her learn more about her emotions and gain insight into her symptoms. She returned to paralegal work and has successfully obtained a therapist to assist her in her continued recovery.

## Discussion

Obsessive-compulsive disorder has been extensively studied and reported as a comorbid condition of psychotic disorders [9]. According to a recent systematic review, psychosis-like symptoms appear to be a common dimension between OCD and psychotic disorders [10]. Others hypothesized that the shared dimension between OCD and psychosis is that of metacognition [11,12], defined as the beliefs, processes, and strategies that monitor, control, or interpret thinking [13]. A cognitive investigation noted that obsessive-compulsive symptoms (OCS) and delusions shared specific metacognitive profiles, particularly through a heightened need to control thoughts [14]. However, OCS is not considered one of the primary manifestations of schizophrenia and is therefore not routinely screened for in patients [15]. It is a recognized challenge to determine if a patient is experiencing delusions (positive symptoms of psychosis) or an OCD obsession (with minimal insight) [16]. In our case, however, undiagnosed OCD further complicated the clinical picture. 

A study by Wahl et al. [17] showed that 70% of the patients who met the diagnostic criteria for OCD did not receive a formal diagnosis or appropriate treatment. Hence, our patient’s initial presentation of attempted suicide, disorganized thought, and bizarre behavior raised the clinical suspicion of first-episode psychosis (FEP). First-episode psychosis in patients diagnosed with OCD has been associated with affective disorder, PDNOS, more depressive symptoms, and higher rates of suicidal plans or attempts at the index point [18]. It was after subsequent in-depth interviews on the inpatient floor that schizo-obsessive spectrum disorder was added to the differential. Schizo-obsessive spectrum disorder is a recently proposed diagnostic term that has gained traction due to research, but it has not been added as an official diagnosis in the DSM. It is characterized by OCD-like symptoms manifesting before the diagnosis of schizophrenia becomes clear. The treating psychiatrist added a low dose of aripiprazole to the treatment regimen and increased the dose of a selective serotonin reuptake inhibitor (SSRI). Aripiprazole has been well-studied as an antipsychotic augmentation agent for SSRI-resistant OCD patients, and there is evidence that second-generation antipsychotics (SGA) with predominant dopaminergic blockade do not induce symptoms of OCS [19]. The psychosis subsided, and our patient's cognition gradually began improving with treatment, which inclined us to believe that the undiagnosed OCD preceded psychosis onset. 

Another important distinction that was drawn retrospectively was the identification of a trigger for the attempted suicide event. The patient did not have obsessive thoughts about self-harm or suicide. She did not have any past history of bodily harm or cutting compulsions. She developed pervasive thoughts and delusional beliefs about her health and financial situation. Coupled with the conviction that these unfounded beliefs were true, she developed suicidal ideation. Mattera et al. [20] suggested a potential evidence-based guide for differentiation between suicidal ideation and suicidal obsessions while describing a case report where the distinction was highlighted by the patient after their own online research about their distressing thoughts. A careful and in-depth review of the psychopathology in the setting of an unclear history revealed a case of undiagnosed OCD presenting with absent insight and ego-dystonic, pervasive obsessive-compulsive beliefs leading to suicidal ideation. This was a rare presentation, and although further studies can further expand existing literature, a singular case cannot be broadly applicable to a wider cohort. One of the strengths of our study was the close collaboration with the patient and her adherence to outpatient follow-up. In terms of future implications, we hope to draw attention to the overlap between OCD and psychotic disorders and highlight the role insight plays in our understanding of these conditions. 

## Conclusions

The association between OCD/OCS and psychotic disorders has been described extensively in the literature and remains widely debated. The possibility of shared pathophysiological bases and multiple overlapping clinical features makes the diagnosis more difficult. Despite the debate, continuing distressing and intrusive thoughts in our patient's case prompted a trial of SGAs in the setting of close monitoring. Although the tailoring of the therapeutic approach is contingent upon establishing the correct diagnosis, a cautious medication trial proved beneficial in our case. The plan had been to discontinue the antipsychotic if the trial showed an unsatisfactory response. However, subsequent improvement in insight led to uncovering of undiagnosed OCD, that was resistant to SSRI. Additionally, our patient was enrolled in a cognitive behavioral therapy program to continue her care. 
